# Quantitative Three-Dimensional Measurements of Acetabular Fracture Displacement Could Be Predictive for Native Hip Survivorship †

**DOI:** 10.3390/jpm12091464

**Published:** 2022-09-06

**Authors:** Anne M. L. Meesters, Miriam G. E. Oldhoff, Neeltje M. Trouwborst, Nick Assink, Joep Kraeima, Max J. H. Witjes, Jean-Paul P. M. de Vries, Kaj ten Duis, Frank F. A. IJpma

**Affiliations:** 1 Department of Surgery, University Medical Center Groningen, University of Groningen, 9713 GZ Groningen, The Netherlands; 2 3D Lab, University Medical Center Groningen, University of Groningen, 9713 GZ Groningen, The Netherlands; 3 Department of Oral and Maxillofacial Surgery, University Medical Center Groningen, University of Groningen, 9713 GZ Groningen, The Netherlands

**Keywords:** acetabular fracture, 3D fracture analysis, 3D gap area, three dimensional, three-dimensional measurements, 3DCT

## Abstract

This study aims to develop a three-dimensional (3D) measurement for acetabular fracture displacement, determine the inter- and intra-observer variability, and correlate the measurement with clinical outcome. Three-dimensional models were created for 100 patients surgically treated for acetabular fractures. The ‘3D gap area’, the 3D surface between all the fracture fragments, was developed. The association between the 3D gap area and the risk of conversion to a total hip arthroplasty (THA) was determined by an ROC curve and a Cox regression analysis. The 3D gap area had an excellent inter-observer and intra-observer reliability. The preoperative median 3D gap area for patients without and with a THA was 1731 mm^2^ versus 2237 mm^2^. The median postoperative 3D gap area was 640 mm^2^ versus 845 mm^2^. The area under the curve was 0.63. The Cox regression analysis showed that a preoperative 3D gap area > 2103 mm^2^ and a postoperative 3D gap area > 1058 mm^2^ were independently associated with a 3.0 versus 2.4 times higher risk of conversion to a THA. A 3D assessment of acetabular fractures is feasible, reproducible, and correlates with clinical outcome. Three-dimensional measurements could be added to the current classification systems to quantify the level of fracture displacement and to assess operative results.

## 1. Introduction

The incidence of acetabular fractures, i.e., fractures involving the hip socket, is estimated as 5 to 8 per 100,000 people per year [1,2]. These fractures can have a serious influence on physical functioning, social activities, and the ability to work. In general, minimally displaced fractures can be treated nonoperatively, and this accounts for approximately half of all acetabular fractures [3,4]. Displaced fractures are mostly treated surgically with reduction and internal fixation; only a small percentage of patients need a primary total hip arthroplasty (THA) [3,5]. The main goal of surgical treatment is to obtain an accurate reconstruction of the articular surface in order to minimize the risk of progressive osteoarthritis and the subsequent need for a THA [6,7]. The residual fracture displacement, measured as the two-dimensional (2D) gap and step-off on computed tomography (CT) slices, is an important factor for estimating the risk for conversion to a THA after surgical treatment of an acetabular fracture. Verbeek et al. reported that an anatomical reduction, according to Matta’s criteria [8] (0–1 mm residual displacement), leads to only 3 percent conversion to a THA, whereas poor reduction (>3 mm residual displacement) leads to 36 percent conversion to a THA after acetabular fractures after a mean follow-up of nine years [9]. They measured the postoperative reduction on radiographs and 2DCT slices. However, these 2D gap and step-off measurements of the initial fracture displacement and postoperative fracture reduction suffer from low inter- and intra-observer agreement [10]. If surgeons still cannot fully agree on the degree of fracture displacement, it will be difficult to assess the results of acetabular fracture surgery and estimate the prognosis by using conventional 2D measurements techniques.

Acetabular fractures usually consist of multiple fracture fragments, which can be displaced in multiple dimensions. The current AO/OTA (Arbeitsgemeinschaft für Osteosynthesefragen/Orthopaedic Trauma Association) classification system only describes the gross fracture pattern but does not include information about the degree of displacement of each fracture fragment [11]. Obtaining insights into the extent of the fracture displacement can be difficult using only 2DCT slices [12]. An understanding of the complexity of the fracture is necessary for determining the treatment strategy, providing the best possible surgical treatment, evaluating the postoperative result, and estimating the prognosis [13]. Three-dimensional imaging and measurements can provide insight into the multidirectional displacement of the fracture fragments and can quantify the true extent of the fracture displacement [14,15,16,17]. Recently, we introduced a 3DCT measurement method for acetabular fractures and compared these measurements with the gold standard 2D gap and step-off measurements [18]. The 3DCT reduction criteria were suggested in previous research [18], but these consist of multiple items, including the 3D gap, 3D step-off, and the total gap area (a 2D surface measurement on a 3D fracture model). Because it is unknown which item is the most important, it can be complicated to decide which criteria must be used in clinical practice and differences between users may occur. Thus, no universal measurement exists that incorporates both the gaps and step-offs between multiple fracture fragments into one measurement. Moreover, the currently available 3DCT measurement method has not been correlated with clinical outcome.

The study did not aim to evaluate the quality of the surgery, but the aim was to test the feasibility of a newly developed measurement method. We hypothesize that a single 3DCT measurement for acetabular fractures will provide an observer-independent analysis of the complexity of the fracture and can be one of the factors that indicate whether a patient is at risk for a THA during follow-up. The aim was to validate our developed 3D measurement method by answering the following research questions: (1) What is the inter- and intra-observer variability of a single 3DCT measurement for the initial and residual displacement in surgically treated acetabular fractures? (2) Is there a relationship between the preoperative 3D measurement and the risk of conversion to a total hip arthroplasty during follow-up? (3) Is there a relationship between the postoperative 3D measurement and the risk of conversion to a total hip arthroplasty in follow-up?

## 2. Materials and Methods

### 2.1. Patients

A diagnostic imaging study was performed in patients treated for acetabular fractures in a level 1 trauma center. Between 2001 and 2020, we treated 428 patients for an acetabular fracture. Of those, we considered surgically treated unilateral acetabular fractures with availability of a high-quality pre- and postoperative CT-scan (with a maximum slice thickness of 2 mm and acquired within four weeks after surgery) and at least one-year clinical follow-up as potentially eligible. Based on that, 63% (270) were eligible; a further 10% (42) were excluded because they were treated with a primary THA (6), were under 18 years old (6), had a periprosthetic fracture (3), had a concomitant pelvic ring injury (24) or a pipkin femoral head fracture (3), and another 30% (128) were deceased (23), were lost prior to the minimum study follow-up of one year (17), or had incomplete datasets (18 patients missing a preoperative CT scan, 45 patients missing a postoperative CT scan, and 25 patients with poor quality CT scans with a slice thickness larger than 2 mm), thus leaving 23% (100) for analysis here. Baseline characteristics were retrieved from the patients’ medical records. All pelvic CT scans at the time of injury were reassessed by two trauma surgeons (KtD, FIJ) and all fractures were classified according to the Letournel classification [19]. All available fracture types were included to prevent potential bias. In our clinic, pre- and postoperative CT scans became standard of care over the past seven years. Before that time, the CT scans were performed based on surgeons’ preferences or indication. Patients were approached by telephone or posted mail and asked whether they received a THA after their acetabular fracture surgery. Indications for conversion to THA were progressive symptomatic osteoarthritis (24/31, Kellgren–Lawrence grade 4 [20]) and avascular necrosis of the femoral head (7/31). Moreover, follow-up information regarding THA was retrieved from the patients’ medical records.

This study was reviewed, and a waiver was provided by the Medical Ethics Review Committee of the University Medical Center Groningen, no: 2016.385. This study is reported following Strengthening the Reporting of Observational Studies in Epidemiology (STROBE) reporting guideline.

### 2.2. Three-Dimensional Measurements

**Figure 1:**
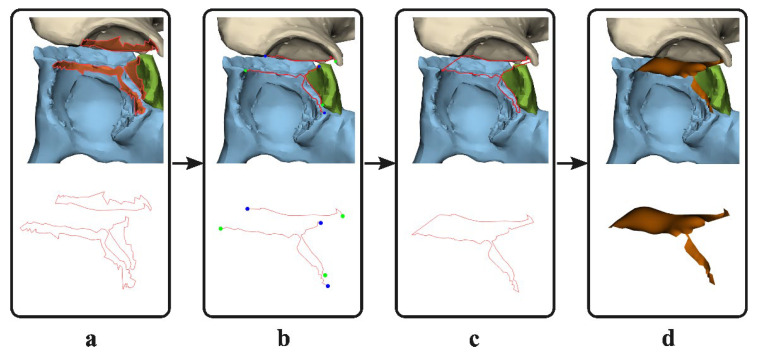
Method for determining the three-dimensional (3D) gap area. (**a**) The surface on the edge of each fracture fragment (e.g., fracture line) is marked (top) and the contours of the surface are converted to curves (red, bottom). (**b**) The curves are cut so that only the fracture lines remain. (**c**) The fracture lines are connected to generate an enclosed curve. (**d**) A three-dimensional surface, the 3D gap area, is generated based on the closed curve of the fracture lines (orange).

Three-dimensional models were created based on the pre- and postoperative CT scans, using the segmentation-certified software of Mimics Medical software (version 19.0; Materialise, Leuven, Belgium). A preset threshold for bone (≥226 Hounsfield Units) was used and all the different fracture fragments were manually separated into individual 3D objects. All 3D objects were imported into the certified 3-matic Medical software (version 13.0; Materialise, Leuven, Belgium). The measurements were first performed on the 3D models derived from the preoperative CT scans. The surface along the edge of the fracture fragments (e.g., the fracture line) was marked (Figure 1a) and separated from the 3D model. The contours of this surface were converted to curves. These curves were trimmed so that the line that remained solely covered the fragments’ fracture edge (Figure 1b). The fracture lines were connected so one enclosed curve was created, which resembled the border of the 3D gap area (Figure 1c). Based on this closed curve, a surface was generated using the surface construction function in 3-matic (Figure 1d). This final generated surface, so called 3D gap area, represents the fracture area (mm^2^) between all fracture fragments. To measure the postoperative 3D gap area, the preoperative fracture fragments were matched with the postoperative 3D model using surface-based matching to avoid the possible influence of metal artefacts. The corresponding preoperative fracture lines were translated together with the fracture fragments and used to determine the 3D gap area postoperatively. All pre- and postoperative 3D models were measured by one observer (AM) experienced in using the 3D software, and it takes on average about two hours per patient to create the 3D models and measure the 3D gap area. Two-dimensional measurements were not included in this study, because previous research showed unreliable results for these measurements [10,18,21].

### 2.3. Statistical Analysis

To answer our first question, regarding the inter- and intra-observer variability of a single 3DCT measurement, twenty pre- and postoperative 3D models were measured by two additional experienced observers (MO, NA). The intraclass correlation coefficient (ICC), with a two-way mixed, single measurements model with absolute agreement, and the 95% confidence interval (CI) were calculated using SPSS (version 23, IBM, Chicago, IL, USA). Moreover, the median difference and interquartile range (IQR) between the values measured by the different observers were calculated. Finally, one observer (AM) repeated all the twenty measurements two times, with an interval of at least one week, and the ICC with 95% CI were calculated to investigate the intra-observer variability.

To answer the second and third question, regarding the relationship between the 3D measurement and the risk of conversion to total hip arthroplasty, the median and IQR were calculated for all continuous data. Frequencies and percentages were calculated for all dichotomous data. The median pre- and postoperative 3D gap area was calculated for all patients. Next, the median pre- and postoperative 3D gap area was calculated for the group of patients with a THA and for the group of patients that retained their native hip. The Mann–Whitney U test was used to compare groups. Finally, a receiver operating characteristics (ROC) curve was created to assess whether the 3D gap area and conversion to THA were related. Critical cut-off values for the pre- as well as postoperative 3D gap area, based on the increased risk of THA, were determined by the value for which the combined sensitivity and specificity is the highest (Youden’s J statistic). These cut-off values were used in a Cox regression analysis for assessing the association between 3D gap area and the risk of conversion to THA and determining a hazard ratio (HR).

## 3. Results

### 3.1. Patients

The median (IQR) age of the included patients was 49 (38–63) years (Table 1). Twenty-eight out of a hundred patients received a THA after a median (IQR) of 16 (11–27) months. Additionally, three patients had an indication for a THA due to symptomatic osteoarthritis (Kellgren–Lawrence grade 4 on follow-up radiograph [20]). They did not receive a THA, because the patients chose to refrain from revision surgery due to comorbidities. These patients were analyzed in the THA group.

### 3.2. Inter- and Intra-Observer Reliability

The inter- and intra-observer reliability was excellent for the pre- and postoperative 3D gap area. For the inter-observer measurements of the 3D gap area, the preoperative ICC was 0.95 (95% CI: 0.84–0.98) and the postoperative ICC was 0.95 (95% CI: 0.89–0.98). The median difference (IQR) between the observers was 182 (102–260) mm^2^ preoperatively and 174 (91–283) mm^2^ postoperatively. For the intra-observer measurements, the preoperative ICC was 0.96 (95% CI: 0.91–0.98) and the postoperative ICC was 0.99 (95% CI: 0.97–0.99). The median difference (IQR) between the repeated measurements was 83 (40–124) mm^2^ preoperatively and 58 (33–115) mm^2^ postoperatively.

### 3.3. Preoperative 3D Measurement Correlated with Clinical Outcome

**Figure 2:**
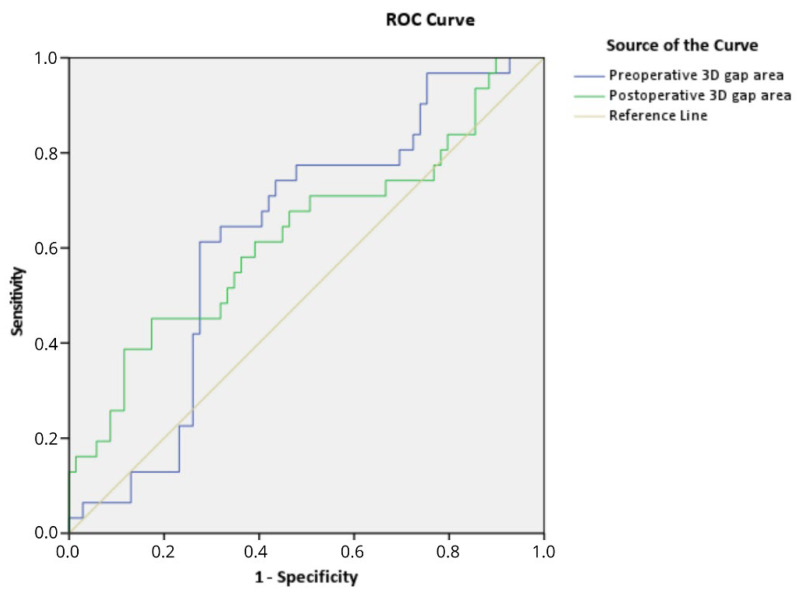
Receiver operating characteristic (ROC) curve demonstrating that the pre- and postoperative 3D gap area are associated with conversion to total hip arthroplasty.

The preoperative 3D gap area is associated with clinical outcome. The overall median (IQR) preoperative 3D gap area for all 100 patients was 1867 (1261–2411) mm^2^. For patients who retained their native hip (N = 69), the median (IQR) preoperative 3D gap area was 1731 (1075–2446) mm^2^ compared to 2237 (1775–2393) mm^2^ (*p* = 0.045) for patients in the THA group (N = 31). The area under the curve was 0.63 (95% CI: 0.51–0.74, *p* = 0.045) for the preoperative 3D gap area (Figure 2). The preoperative critical cut-off value for a conversion to a THA was 2103 mm^2^ with a sensitivity of 61% and a specificity of 73%. The Cox regression analysis, adjusted for age and gender, showed that a preoperative 3D gap area > 2103 mm^2^ (critical cut-off) was independently associated with a 3.0 times higher risk of conversion to a THA (adjusted: HR 3.0, 95% CI: 1.4–6.2, *p* = 0.004; unadjusted: HR 3.1, 95% CI: 1.5–6.4, *p* = 0.002).

### 3.4. Postoperative 3D Measurement Correlated with Clinical Outcome

**Figure 3:**
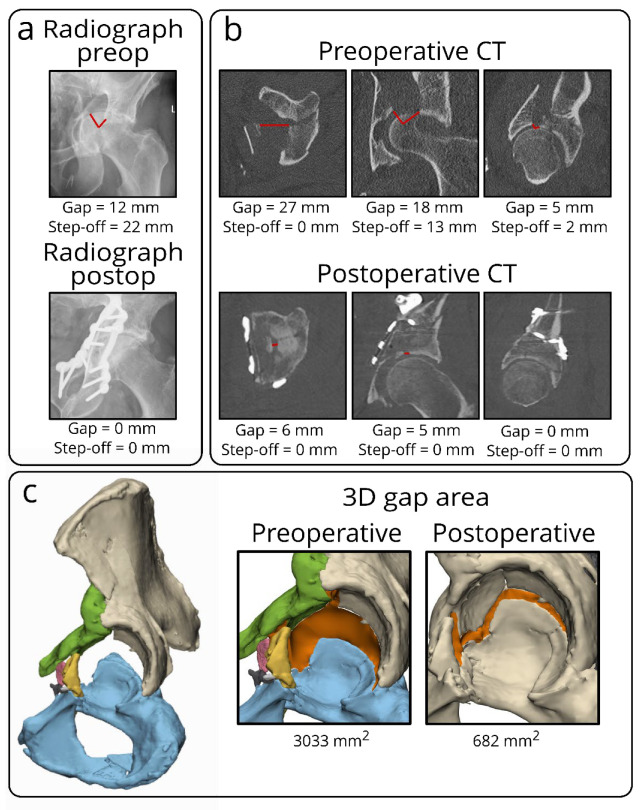
Case example of a both-column fracture in a 63-year-old male, showing the discrepancy in measuring initial and residual fracture displacement for acetabular fractures on different imaging modalities, including radiographs, CT scans, and 3D models. On radiographs (**a**), it is difficult to measure gaps and step-offs, especially on the postoperative radiograph, because the implant is partially obscuring the acetabulum. On the single CT slices (**b**), multiple gaps and step-offs (red lines) can be measured on different CT slices in several planes, indicating the subjective elements of these measurements. The 3D model (**c**) demonstrates the 3D gap area (in orange) representing the three-dimensional surface between all fracture fragments. This should be considered a single quantitative measure of the initial or residual fracture displacement in the entire acetabulum.

**Figure 4:**
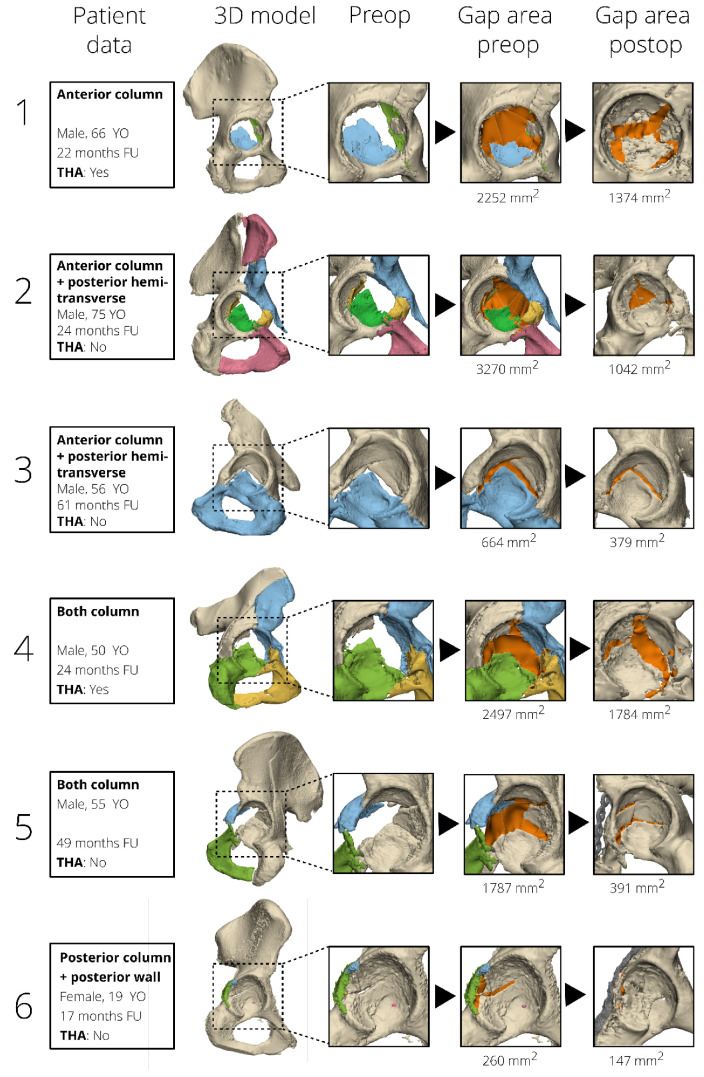

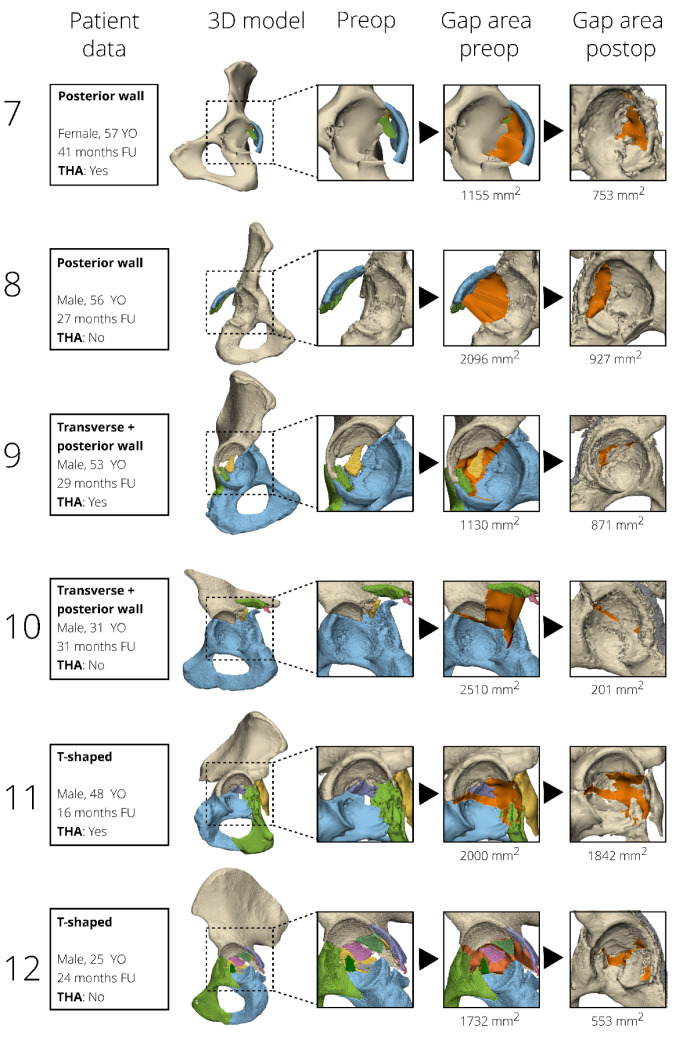
Case examples of 12 patients, surgically treated for different types of acetabular fractures, are presented in order to correlate fracture displacement (as measured by the 3D gap area, orange) to clinical outcome. The pre- and postoperative 3D gap area are indicated in orange. The cases are sorted based on fracture type. YO: years old, FU: follow-up, THA: total hip arthroplasty.

The postoperative 3D gap area is associated with clinical outcome. The overall median (IQR) postoperative 3D gap area for all 100 patients was 679 (310–1074) mm^2^. The median (IQR) postoperative 3D gap area was 640 (311–961) mm^2^ for patients who retained their native hip, compared to 845 (298–1456) mm^2^ for patients in the THA group (*p* = 0.045). For the postoperative 3D gap area, the area under the curve was 0.63 (95% CI: 0.50–0.75, *p* = 0.045). The postoperative critical cut-off value was 1058 mm^2^ with a sensitivity of 45% and a specificity of 83% for a conversion to a THA. The Cox regression analysis, adjusted for age and gender, showed that a postoperative 3D gap area > 1058 mm^2^ (critical cut-off) was independently associated with a 2.4 times higher risk of conversion to a THA (adjusted: HR 2.4, 95% CI: 1.1–5.2, *p* = 0.021; unadjusted: HR 2.7, 95% CI: 1.3–5.5, *p* = 0.006). The clinical case examples of the 3D gap area are shown in Figure 3 and Figure 4, Figures S1 and S2.

## 4. Discussion

The conventional 2DCT single slice gap and step-off measurements of an initial fracture displacement and postoperative fracture reduction, which are currently used to evaluate the results of acetabular fracture surgery, suffer from low inter- and intra-observer agreement and do not represent the displacement of all fracture fragments [10]. The aim of this study was to develop and evaluate our single 3DCT measurement in order to quantify the preoperative fracture displacement and postoperative reduction in acetabular fracture surgery by determining the inter- and intra-observer variability and investigating the relationship between the 3D measurement and the risk of conversion to a THA. We introduced the 3D gap area measurement that represents the 3D surface area between all fracture fragments. The measure was developed and assessed on the pre- and postoperative 3D models of 100 patients. Our study shows that the 3D gap area can be reliably measured and accurately reproduced, with high inter- and intra-observer reliability. The 3D gap area measurements represent an observer-independent single quantitative measure for assessing the initial fracture displacement and postoperative fracture reduction. Patients who needed a THA had a higher median pre- and postoperative 3D gap area compared to those who retained their native hip, indicating that the 3D gap area is associated with clinical outcome.

This study contains several limitations. First of all, the 3D software, 3D expertise, and manpower needed for the measurements are not available in all hospitals. Second, there is a selection bias, because only the patients with a postoperative CT scan were included. These postoperative CT scans were only made standard of care over the last 7 years, whereas before this time, a postoperative CT scan was only performed upon indication. However, this does not affect our research method, because our study aimed to introduce and assess a 3D measurement technique and link those to the risk of conversion to a THA instead of reporting on the clinical outcome itself. Finally, creating 3D models and performing the 3D gap area measurements is time-consuming and will take on average two hours per patient. Yet, future developments in software might reduce this time, making it more applicable in clinical practice.

Accuracy and reproducibility of measurements for assessing fracture displacement is mandatory to use them with confidence in clinical practice. In this study, we introduced a new 3D measurement for assessing acetabular fracture displacement that is accurate, reliable, and does not depend on the subjective interpretations of surgeons. The gap and step-off measurements using traditional 2D imaging (e.g., radiographs or 2DCT slices) have proven to be insufficient for assessing the displacement of acetabular fractures [10,21]. The assessment of a fracture relies on where the 2D measurement is performed, meaning which fracture line or CT slice is selected for the measurement and how the measurement is performed. A previous study demonstrated a low inter-observer reliability of the gap and step-off measurements on 2DCT slices, with ICCs varying from 0.3–0.4 [10]. The unique feature of our new 3D gap area measurement is that it includes the entire fractured area and combines the gaps and step-offs between all fracture fragments in one 3D surface. This approach enables expressing the degree of initial and residual fracture displacement in a single quantitative measure for the first time.

Quantifying the initial fracture displacement and postoperative reduction is essential for the treatment decision and patient counseling regarding the prognosis. The 3D gap area was correlated with conversion to a THA in order to assess whether it could potentially be used as a predictive value for the clinical outcome. The median initial displacement (preoperative 3D gap area) and the median residual displacement (postoperative 3D gap area) were higher in the group of patients that received a THA during follow-up, indicating that the 3D gap area is associated with clinical outcome. Moreover, if the preoperative 3D gap area can be used to predict the risk of conversion to a THA during follow-up, this could have major implications in deciding about osteosynthesis versus a primary THA at the time of the injury. This study provides a preliminary critical cut-off value for the preoperative 3D gap area (>2103 mm^2^; HR 3.0, 95% CI 1.4–6.2; *p* = 0.004), which is independently associated with the risk of conversion to a THA. Obviously, definitive cut-off values for relating the 3D gap area to the risk of conversion to a THA still need to be determined in a larger series. On the other hand, we noticed that the pre- and postoperative 3D gap area does not always correlate with clinical outcome. For instance, one patient with a relatively small pre- and postoperative 3D gap area (625 and 205 mm^2^, e.g., indicating limited initial displacement and proper fracture reduction) eventually received a THA due to avascular necrosis of the femoral head instead of due to progressive osteoarthritis caused by residual fracture displacement. In this study, pre- and postoperative 3D gap areas still have moderate discriminating ability (area under the curve of 0.63) between whether or not a conversion to a THA will be needed at follow-up. This might be explained by the fact that multiple patient factors, including age, comorbidity, femoral head injuries, and dome impaction, are associated with clinical outcome as well. Another important parameter could be the location of the fracture displacement, e.g., a fracture of the weightbearing dome might be more likely to cause osteoarthritis and conversion to a THA. A larger multicenter follow-up study, including both patient as well as fracture characteristics, is needed to unravel the true discriminating ability of the 3D gap area. Overall, the introduction of our 3D gap area for assessing the fracture displacement should be considered as a first step away from the traditional observer-dependent 2D gap and step-off measurements and toward a new era of a standardized advanced 3D evaluation of operative results. The 3D technology for acetabular fracture surgery has been increasingly used around the world in the past few years [22]. We envision that an automatic segmentation and (semi-)automated 3D analysis of fracture displacement will be possible in the near future. For instance, the 3D gap area measurement could be integrated in commercially available surgical planning software in order to standardize the evaluations of the operation results and eventually estimate prognosis.

The 3D gap area measurement represents a reproducible single measurement to quantify the initial fracture displacement and postoperative fracture reduction in acetabular fracture treatment. The unique aspect of this measurement is that it includes the entire fractured area and incorporates gaps and step-offs between all fracture fragments in one 3D surface. Moreover, it is a single quantitative measure that is associated with clinical outcome. In general, we envision that 3D measurements of fracture displacement will open a new era of evaluating operation results.

## Figures and Tables

**Table 1:** Patient characteristics. THA: Total hip arthroplasty.

Patient Demographics (N = 100)			
	Native Hip (N = 69)	THA (N = 31)	Total
**Sex (no.)**			
Male	59	24	83
Female	10	7	17
**Median (IQR) age (in years)**	48 (34–62)	53 (41–67)	49 (38–64)
**Letournel classification (no.)**			
Anterior column	5	1	6
Posterior column	1	0	1
Posterior wall	15	6	21
Transverse	1	0	1
Anterior column and posterior hemitransverse	6	0	6
Both column	23	9	32
Posterior column and posterior wall	4	3	7
T-type	8	3	11
Transverse and posterior wall	6	9	15

## Data Availability

The authors declare that the data supporting the findings of this study are available within the paper.

## Supplementary Materials

The following supporting information can be downloaded at: https://www.mdpi.com/article/10.3390/jpm12091464/s1, **Figure S1:** Pre- and postoperative 3D gap area. Three-dimensional gap area measurement (indicated in orange) showing the preoperative (initial) and postoperative (residual) fracture displacement of a 39-year-old woman with a transverse-posterior wall type of acetabular fracture. The preoperative 3D gap area was 1251 mm^2^ and the postoperative 3D gap area was 324 mm^2^. **Figure S2:** Both-column examples. Case examples of four patients, surgically treated for both-column acetabular fractures, are presented in order to visually correlate fracture displacement (as measured by the 3D gap area) to clinical and radiological outcome. The pre- and postoperative 3D gap area is indicated in orange. Patients who had conversion to THA at follow-up had osteoarthritis on the follow-up radiograph. YO: years old, FU: follow-up, THA: total hip arthroplasty.

## References

[1] Rinne P.P., Laitinen M.K., Huttunen T., Kannus P., Mattila V.M. (2017). The incidence and trauma mechanisms of acetabular fractures: A nationwide study in Finland between 1997 and 2014. Injury.

[2] Melhem E., Riouallon G., Habboubi K., Gabbas M., Jouffroy P. (2020). Epidemiology of pelvic and acetabular fractures in France. Orthop. Traumatol. Surg. Res..

[3] Boudissa M., Francony F., Kerschbaumer G., Ruatti S., Milaire M., Merloz P., Tonetti J. (2017). Epidemiology and treatment of acetabular fractures in a level-1 trauma centre: Retrospective study of 414 patients over 10 years. Orthop. Traumatol. Surg. Res..

[4] Clarke-Jenssen J., Wikerøy A.K., Røise O., Øvre S.A., Madsen J.E. (2016). Long-term survival of the native hip after a minimally displaced, nonoperatively treated acetabular fracture. J. Bone Jt. Surg. Am..

[5] Grubor P., Krupic F., Biscevic M., Grubor M. (2015). Controversies in treatment of acetabular fracture. Med. Arch. (Sarajevo, Bosnia Herzegovina).

[6] Verbeek D.O., van der List J.P., Tissue C.M., Helfet D.L. (2018). Long-term patient reported outcomes following acetabular fracture fixation. Injury.

[7] Giannoudis P.V., Grotz M.R.W., Papakostidis C., Dinopoulos H. (2005). Operative treatment of displaced fractures of the acetabulum. A meta-analysis. J. Bone Jt. Surgery. Br. Vol..

[8] Matta J.M. (1996). Fractures of the acetabulum: Accuracy of reduction and clinical results in patients managed operatively within three weeks after the injury. J. Bone Joint Surg. Am..

[9] Verbeek D.O., Van Der List J.P., Villa J.C., Wellman D.S., Helfet D.L. (2017). Postoperative CT is superior for acetabular fracture reduction assessment and reliably predicts hip survivorship. J. Bone Jt. Surg. Am. Vol..

[10] Meesters A.M.L., ten Duis K., Banierink H., Stirler V.M.A., Wouters P.C.R., Kraeima J., De Vries J.-P.P.M., Witjes M.J.H., Ijpma F.F.A. (2020). What are the interobserver and intraobserver variability of gap and stepoff measurements in acetabular fractures?. Clin. Orthop. Relat. Res..

[11] Meinberg E.G., Agel J., Roberts C.S., Karam M.D., Kellam J.F. (2018). Fracture and dislocation classification compendium—2018. J. Orthop. Trauma.

[12] Scheinfeld M.H., Dym A.A., Spektor M., Avery L.L., Dym R.J., Amanatullah D.F. (2015). Acetabular fractures: What radiologists should know and how 3D CT can aid classification. RadioGraphics.

[13] Matta J.M. (1986). Operative indications and choice of surgical approach for fractures of the acetabulum. Technol. Orthop..

[14] Mellema J.J., Janssen S.J., Guitton T., Ring D. (2015). Quantitative 3-dimensional computed tomography measurements of coronoid fractures. J. Hand Surg..

[15] Lubberts B., Janssen S., Mellema J., Ring D. (2015). Quantitative 3-dimensional computed tomography analysis of olecranon fractures. J. Shoulder Elb. Surg..

[16] De Muinck Keizer R.-J.O., Meijer D.T., van der Gronde B.A.T.D., Teunis T., Stufkens S.A.S., Kerkhoffs G.M., Goslings J.C., Doornberg J.N. (2016). Articular gap and step-off revisited: 3D quantification of operative reduction for posterior malleolar fragments. J. Orthop. Trauma.

[17] Assink N., Kraeima J., Slump C.H., Duis K.T., De Vries J.P.P.M., Meesters A.M.L., van Ooijen P., Witjes M., Ijpma F.F.A. (2019). Quantitative 3D measurements of tibial plateau fractures. Sci. Rep..

[18] Meesters A.M.L., Kraeima J., Banierink H., Slump C.H., De Vries J.P.P.M., ten Duis K., Witjes M.J.H., Ijpma F.F.A. (2019). Introduction of a three-dimensional computed tomography measurement method for acetabular fractures. PLoS ONE.

[19] Tile M., Helfet D.L., Kellam J.F., Vrahas M. (2015). Fractures of the Pelvis and Acetabulum—Principles and Methods of Management.

[20] Kellgren J., Lawrence J. (1957). Radiological assessment of osteo-arthrosis. Ann. Rheum. Dis..

[21] Meesters A.M.L., ten Duis K., Kraeima J., Banierink H., Stirler V.M.A., Wouters P.C.R., de Vries J.P.P.M., Witjes M.J.H., IJpma F.F.A. (2021). The accuracy of gap and step-off measurements in acetabular fracture treatment. Sci. Rep..

[22] Meesters A.M.L., Trouwborst N.M., De Vries J.P.M., Kraeima J., Witjes M.J.H., Doornberg J.N., Reininga I.H.F., IJpma F.F.A., ten Duis K. (2021). Does 3D-Assisted Acetabular Fracture Surgery Improve Surgical Outcome and Physical Functioning ?— A Systematic Review. J. Pers. Med..

