# Supplementary figures and images for: Quantitative Three-Dimensional Measurements of Acetabular Fracture Displacement Could Be Predictive for Native Hip Survivorship
Source: J Pers Med. 2022 Sep 6;12(9):1464. doi: 10.3390/jpm12091464 (PMC9501648; doi:10.3390/jpm12091464)

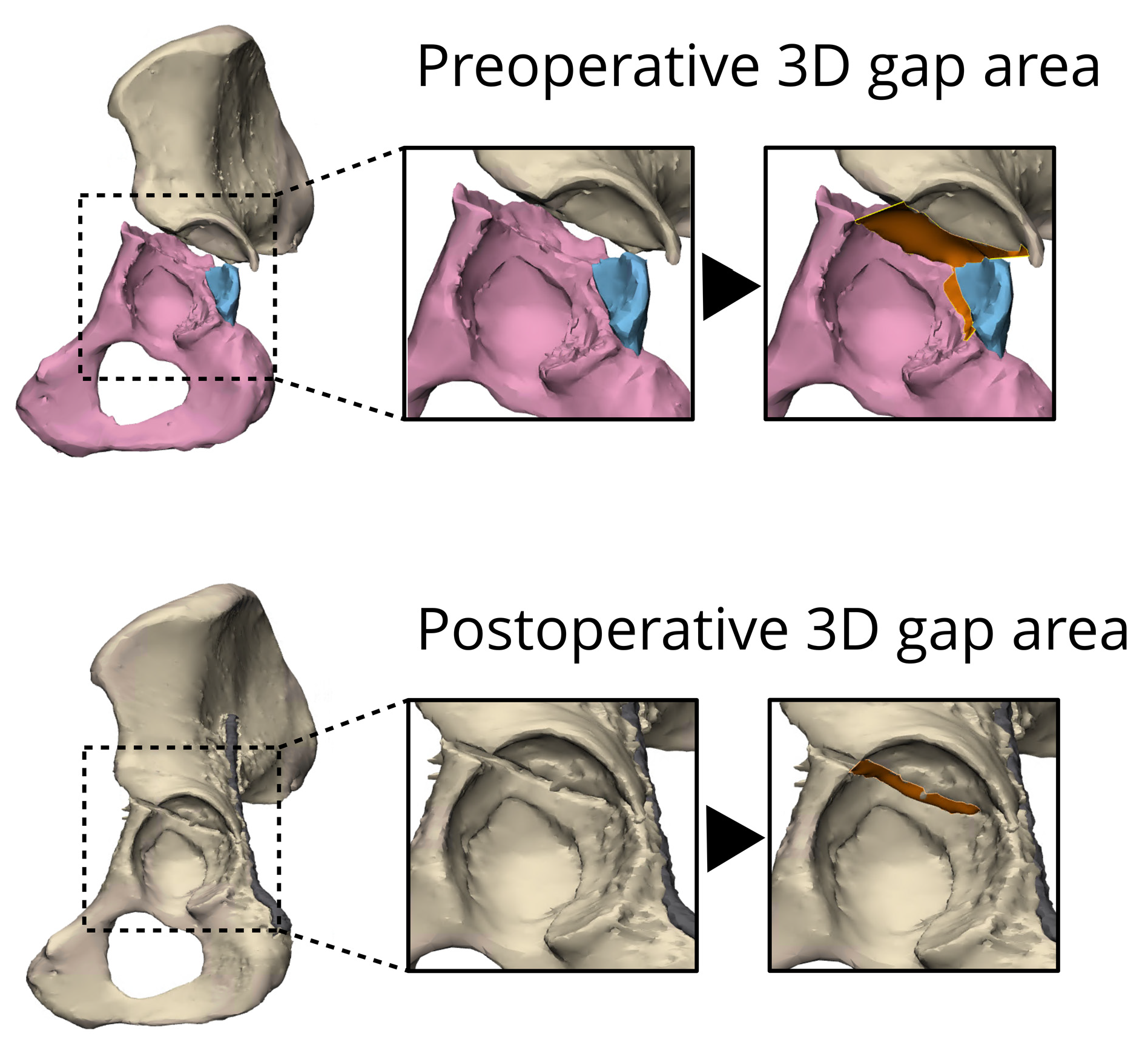

Supplement: Supplementary file 1 [file jpm-12-01464-s001.zip › Figure S1.png]
